# BPhyOG: An interactive server for genome-wide inference of bacterial phylogenies based on overlapping genes

**DOI:** 10.1186/1471-2105-8-266

**Published:** 2007-07-25

**Authors:** Yingqin Luo, Cong Fu, Da-Yong Zhang, Kui Lin

**Affiliations:** 1 MOE Key Laboratory for Biodiversity Science and Ecological Engineering and College of Life Sciences, Beijing Normal University, Beijing 100875, China

## Abstract

**Background:**

Overlapping genes (OGs) in bacterial genomes are pairs of adjacent genes of which the coding sequences overlap partly or entirely. With the rapid accumulation of sequence data, many OGs in bacterial genomes have now been identified. Indeed, these might prove a consistent feature across all microbial genomes. Our previous work suggests that OGs can be considered as robust markers at the whole genome level for the construction of phylogenies. An online, interactive web server for inferring phylogenies is needed for biologists to analyze phylogenetic relationships among a set of bacterial genomes of interest.

**Description:**

BPhyOG is an online interactive server for reconstructing the phylogenies of completely sequenced bacterial genomes on the basis of their shared overlapping genes. It provides two tree-reconstruction methods: Neighbor Joining (NJ) and Unweighted Pair-Group Method using Arithmetic averages (UPGMA). Users can apply the desired method to generate phylogenetic trees, which are based on an evolutionary distance matrix for the selected genomes. The distance between two genomes is defined by the normalized number of their shared OG pairs. BPhyOG also allows users to browse the OGs that were used to infer the phylogenetic relationships. It provides detailed annotation for each OG pair and the features of the component genes through hyperlinks. Users can also retrieve each of the homologous OG pairs that have been determined among 177 genomes. It is a useful tool for analyzing the tree of life and overlapping genes from a genomic standpoint.

**Conclusion:**

BPhyOG is a useful interactive web server for genome-wide inference of any potential evolutionary relationship among the genomes selected by users. It currently includes 177 completely sequenced bacterial genomes containing 79,855 OG pairs, the annotation and homologous OG pairs of which are integrated comprehensively. The reliability of phylogenies complemented by annotations make BPhyOG a powerful web server for genomic and genetic studies. It is freely available at .

## Background

With the increasing availability of genome sequences, methods using vast amounts of phylogenetic information contained in complete genome sequences are becoming standard for inferring species phylogenies reliably. Because phylogenetic markers extracted from whole genome resource are based on the maximum genetic information, a phylogenetic tree should be the best reflection of the evolutionary history of the species [1,2]. To our knowledge, the evolutionary distance between two different genomes defined by extant phylogenetic markers – such as gene content and gene order – may not be suitable for inferring large-scale evolutionary relationships among microbial genomes, because gene contents have changed too little and gene order has changed too much [3]. We previously reported that overlapping genes can provide interesting additional insights into phylogenetic relationships [4]. As phylogenetic markers, on the one hand, OGs evidently do not evolve slowly as gene content does because they are rampant in prokaryotic genomes, even varying largely among closely-related genomes; on the other hand, OGs show more evolutionary conservation than gene order because functional constraints may preserve the linkage between two overlapping genes [3,5-7]. Indeed, OGs are consistent present in all microbial genomes that have been sequenced to date [8,9]. Therefore, the evolution of these sequences is probably related to the evolutionary timescale, so they can be used to reconstruct phylogenies [4].

In addition to their suitability as markers for reconstructing phylogenies, OGs are considered very important for studying the evolution of prokaryotic genomes. Since the first OGs was discovered in 1976 in bacteriophage *phiX174 *[10], the traditional view has changed; it is now accepted that different open reading frames (ORFs) can share common coding regions. Two main evolutionary explanations of this important phenomenon have been suggested. Some authors view it as a means of compressing the maximum amount of information into shorter sequences of structural genes; thus, it may be a result of evolutionary pressure to minimize genome size and increase the density of genetic information. Others argue that it might be a mechanism for regulating gene expression through translational coupling of functionally related polypeptides [11-14].

Overlaps have also been shown to be potentially important in transcriptional and translational regulation of gene expression and to influence the evolution of genes [15]. In prokaryotic genomes, unidirectional (→→) neighbors are most widely conserved and it is generally accepted that conserved operons strongly indicate functional associations [9,16,17], so links can be predicted between all conserved OG pairs. Also, if two overlapping genes are divergently (←→) transcribed with conserved gene orientation, they must be strongly co-regulated [13]. Thus, a simple and friendly web interface that can provide users with a convenient way to acquire useful information about OGs is very desirable.

To satisfy these requirements and to provide an easy-to-use platform for phylogenetic inferences, we have developed BPhyOG. This is an interactive web server that can be used to infer bacterial phylogenies for a set of completely sequenced genomes of interest. It also allows users to browse the OGs used to infer the phylogenetic relationships, or all the OGs from 177 genomes, and provides comprehensive annotation and homologous OG pairs on a large scale. BPhyOG is freely available as an online service and addresses many of the needs described above. The results of BPhyOG are displayed in easy-to-understand tabulated and graphical formats.

## Construction and content

### Data resources

Bacterial genomes without plasmids were downloaded in the GenBank format from the NCBI ftp server in August 2004. A total of 79,855 overlapping gene pairs were extracted from the 177 acquired bacterial genomes using C/C++ scripts. The data are stored in a MySQL database.

### Retrieval and identification of overlapping genes

Overlapping genes are defined as adjacent genes, on either strand, that have coding sequences (CDS regions) sharing one or more bases. They were extracted from each genome using PERL scripts according to the annotations. OG pairs were classified into three directional patterns: 'convergent' (→←), 'unidirectional' (→→) and 'divergent' (←→). 'Overlap phase type' reflects the corresponding codon positions that the overlap segment takes in each of the individual overlapping genes. For example, the overlap phase type of the OG pair '*83333_7*' (<*yabF*, *kefC*>) is denoted '<2:3, 1:2>', which means that in gene *yabF*, the initial position of the overlap segment is the second codon position and the terminal position is the third codon position; and in *kefC*, the initial and terminal positions are the first and second codon positions, respectively. As we know, misannotated open reading frames (ORFs) may be included in the genome data. Therefore, to improve the accuracy of prediction, we defined conserved homologous OG pairs as those for which the products of both overlapped genes are not annotated as "*hypothetical*" or "*putative*" or "*unknown*". However, we still listed all the OG pairs of any given genome for other possible uses such as reannotation.

### Definition of orthologous overlapping genes and reconstruction of phylogenies

In our work, we used NCBI BLAST version 2.2.6 [Apr-09-2003, for Linux IA-64 systems] [18] to look for possible orthologous genes among the 177 genomes by searching bidirectional best hits and applying thresholds of e-value < 10^-4 ^and identity >40%. We then defined orthologous OG pairs from two different genomes as pairs of genes that overlap in genome *i *and have orthologous counterparts that overlap in genome *j *[4].

The distance between two genomes is defined as:

Dij=1−xij+xji2∗min⁡(xi,xj)i,j=1,2,...N
 MathType@MTEF@5@5@+=feaafiart1ev1aaatCvAUfKttLearuWrP9MDH5MBPbIqV92AaeXatLxBI9gBaebbnrfifHhDYfgasaacH8akY=wiFfYdH8Gipec8Eeeu0xXdbba9frFj0=OqFfea0dXdd9vqai=hGuQ8kuc9pgc9s8qqaq=dirpe0xb9q8qiLsFr0=vr0=vr0dc8meaabaqaciaacaGaaeqabaqabeGadaaakeaafaqabeqacaaabaGaemiraq0aaSbaaSqaaiabdMgaPjabdQgaQbqabaGccqGH9aqpcqaIXaqmcqGHsisldaWcaaqaaiabdIha4naaBaaaleaacqWGPbqAcqWGQbGAaeqaaOGaey4kaSIaemiEaG3aaSbaaSqaaiabdQgaQjabdMgaPbqabaaakeaacqaIYaGmcqGHxiIkcyGGTbqBcqGGPbqAcqGGUbGBcqGGOaakcqWG4baEdaWgaaWcbaGaemyAaKgabeaakiabcYcaSiabdIha4naaBaaaleaacqWGQbGAaeqaaOGaeiykaKcaaaqaaiabdMgaPjabcYcaSiabdQgaQjabg2da9iabigdaXiabcYcaSiabikdaYiabcYcaSiabc6caUiabc6caUiabc6caUiabd6eaobaaaaa@5817@

where *x*_*i *_is the number of OG pairs in genome *i*, *N *is the number of selected species and *x*_*ij *_is the number of OG pairs in genome *i *with orthologs in genome *j*. From this equation, an N × N distant matrix is produced, which indicates the evolutionary relationship among the selected N genomes based on the OGs (see [4] for details). Users can then choose either of the tree-reconstructing methods, NJ [19] or UPGMA [20], to infer a phylogeny for the selected genomes.

## Utility

We developed an interactive web server named BPhyOG, which currently contains 177 completely sequenced bacterial genomes. It facilitates reconstruction of whole genome phylogenies and also allows users to browse OGs to acquire more information about subtree-specific genomes or all the 177 genomes. Basically, it comprises three sections: (i) phylogenetic inference, which is mainly used to reconstruct phylogenies for a set of genomes of interest; (ii) OG pairs browse, which allows users to retrieve annotated information about OG pairs for further functional or evolutionary studies; and (iii) searching interface.

### Phylogenetic inference

**Figure 1:**
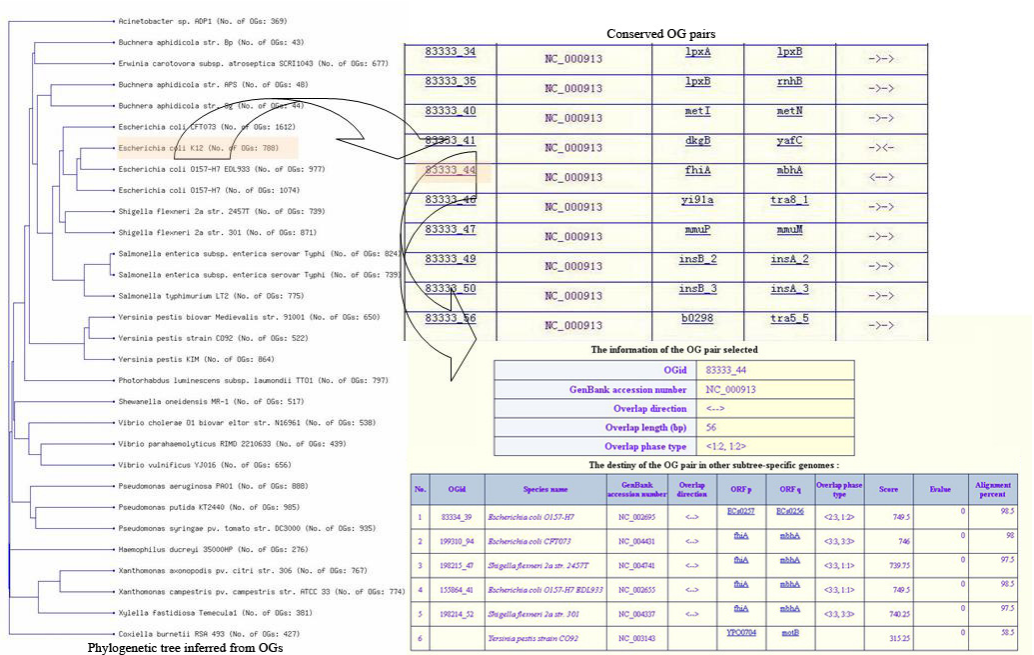
**Flow chart of the phylogenetic tree of 30 gamma-proteobacterial genomes reconstructed on the basis of conserved OG pairs**. The left panel shows a phylogenetic UPGMA tree for the 30 gamma-proteobacterial genomes based on the number of their shared OG pairs. Each branch is labeled by the species name and the total number of OG pairs in the genome in square brackets. The right upper panel shows all the conserved OG pairs in the genome of interest when the species name is clicked on the tree, e.g. *Escherichia coli K12*. The right lower panel describes the information about one OG pair (e.g. *83333_44*) and the list of its orthologous OG pairs within the subtree-specific genomes.

BPhyOG allows users to infer the phylogenetic relationships among a set of genomes on the basis of the number of orthologous OG pairs; the inferred tree is directly visualized online. It provides two methods for reconstructing phylogenetic trees: NJ, a general method; and UPGMA. The latter is more suitable for inferring phylogenies when the rate of evolution of the phylogenetic marker is relatively constant [21]. Clicking the "Phylogeny Inference" box shows all 177 genomes listed in a table. When a set of genomes of interest is selected with a preferred method, a phylogenetic tree for those genomes can be inferred and displayed on a new page. As an example, Fig. 1 shows a phylogenetic tree for 30 gamma-proteobacteria genomes selected from the table. When the species name is clicked on the tree visualized on the web, detailed information about the OG pairs used to infer the phylogenetic relationships is displayed on a new page. Users can then exploit the evolutionary relationship among two overlapped genes in other subtree-specific genomes to see whether the linkage has been broken or whether they still overlap. The inferred phylogenetic tree can also be downloaded in Newick format for further study.

### OG pairs browse

**Figure 2:**
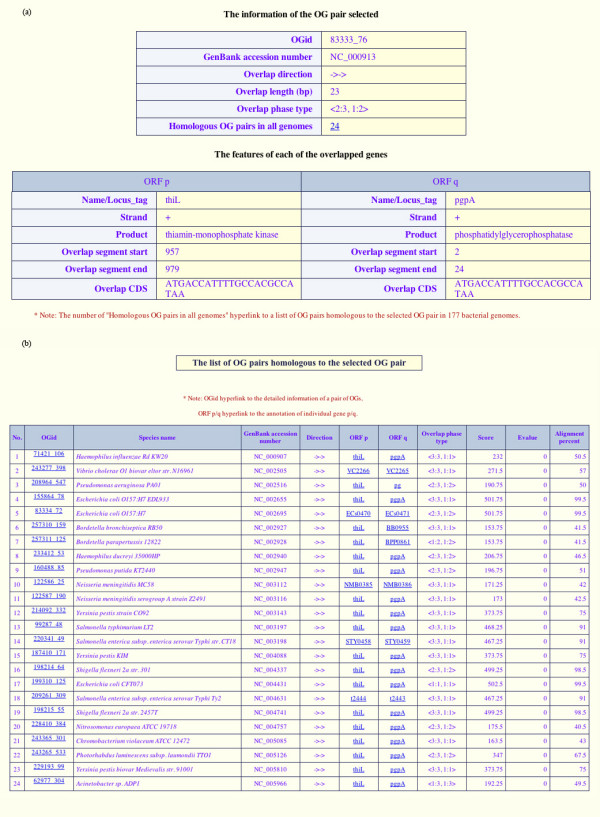
**The detailed features of one OG pair as an example, and a list of homologous OG pairs in all genomes**. (a) The upper table describes the information about a selected OG pair, and the lower one lists details of the overlap features of each gene in the pair. (b) The list of predicted OG pairs homologous to the OG pair selected (OGid *83333_76*) among all genomes. Two homologous OG pairs from two different genomes (g_*i*_, g_*j*_) are defined as two genes that overlap in genome g_*i *_and have homologues that overlap in genome g_*j*_.

Users can browse all OG pairs in a genome, or a particular OG pair of interest in two ways: through hyperlinks or by manual searching. The first page obtained by clicking the "OGs in Genomes" box displays a summary of the 177 bacterial genomes. This page gives basic features of the genomes including the species name, GenBank accession number, whole genome sequence size, coding sequence size, number of ORFs, and number of OG pairs. On clicking the species name, a list of OG pairs with individual overlapping genes and overlap direction (→←, →→ and ←→) is displayed on a new page. Each "OGid" leads to detailed information about the corresponding OG pair on a new page, which comprises two frames (Fig. 2a). The upper frame lists survey information about the OG pair, including the number of homologous OG pairs found in all genomes and the number of links to the list of homologous OG pairs in other genomes (Fig. 2b). Conversely, all links in the list of homologous OG pairs are provided to retrieve the description of OG pair and the annotation of individual genes. The lower frame presents features of the two overlapping genes such as the start, the end and the nucleotide sequence of the overlap segment (Fig. 2a).

### Searching interface

The searching interfaces are convenient and enable users to acquire information in several ways. Users can query whether a gene overlaps with others by entering its name, or by entering its name and GenBank accession number together. They can also retrieve a list of OG pairs from a genome of interest quickly, by entering a GenBank accession number or directly selecting a species name from the scrolling list. The query results are displayed in tabular form on a new page and users can study detailed information through further hyperlinks. All hyperlinks are noted carefully as query assistants.

## Discussion

### Benchmarking the phylogenies inferred from OG pairs

The overall survey of phylogenies determined by OGs is likely to provide an accurate glimpse of the "big picture" of the tree of life for bacteria. It suggests that the number of OGs in each bacterial genome is commensurate with the number of ORFs (see Additional file 1). Fukuda *et al*. and Johnson *et al*. also showed that the evolution of overlapping gene structures may be related to the evolutionary time scale [7-9]. Therefore, assuming a universal rate of formation and degradation of OGs across species, we can determine the evolutionary distance between two bacterial genomes on the basis of the number of their shared OG pairs. We designed an interactive web server, BPhyOG, to reconstruct whole genome phylogenies on the basis of overlapping genes.

BPhyOG provides users with two methods to infer phylogenies, namely UPGMA and NJ. As an example, we selected 30 gamma-proteobacteria. The left hand side of Fig. 1 shows the phylogenetic tree inferred by UPGMA, as visualized on the webpage, for the 30 gamma-proteobacteria genomes. Benchmarking the UPGMA tree with the tree based on 16S rRNA sequences (see Additional file 2, (a) for a simple 16S rRNA tree of these taxa, and (b) for the OG tree generated using UPGMA; the Newick format treefile was downloaded from the webpage), two tree topologies were derived that differed mainly in the positions of three endosymbionts (*Buchnera aphidicola str. Sg*, *B. aphidicola str. APS *and *B. aphidicola str. Bp*), *Escherichia coli K12 *and *Haemophilus ducreyi*. Our tree based on OG pairs considered the four *E. coli *strains to be monophyletic, whereas the 16S rRNA tree placed the *E. coli K12 *strain closer to *Shigella flexneri*. This demonstrates that OGs as phylogenetic markers offer high taxonomic resolution. In addition, our OG tree confirms that the *Buchnera *order is a sib-group of *E. coli *and *Salmonella *rather than to *Yersinia pestis*, which is consistent with previous studies [22,23]. However, *Haemophilus *was placed as a sib of the *Pseudomonas *order, which does not agree with current classifications. This may be because the genome of *Haemophilus *is too small for our measure to obtain an accurate evolutionary distance.

### Tracking the conservation of OG pairs used to infer phylogenetic relationships

Besides inferring phylogenetic trees for whole genomes, BPhyOG provides an easy-to-use interface for users to track the conservation of OG pairs by exploring homologous pairs in subtree-specific genomes. As a case study, the phylogeny of 30 gamma-proteobacteria genomes was inferred and visualized on the webpage. When the species name, such as *Escherichia coli K12*, was clicked, a set of conserved OG pairs was listed in a table (the right upper panel of Fig. 1). Then, by clicking on any "OGid" of interest, e.g. "*83333_44*" (<*fhiA*, *mbhA*>), a new page with two frames was opened. The top frame showed the features of the queried OG pair "*83333_44*" and the lower frame showed the list of OG pairs homologous to "*83333_44*" in the subtree-specific genomes (Fig. 1). From the list in the lower frame, we can see that the two overlapping genes (*fhiA *and *mbhA*) in "*83333_44*" still overlap in three other *E. coli *strains (NC_002695, NC_004431 and NC_002600) and in two *Shigella flexneri *strains(NC_004741 and NC_004337), whereas the linkage has been broken in *Yersinia pestis strain CO92 *(NC_000913) and lost in the remaining 23 gamma-proteobacteria genomes.

In summary, BPhyOG is a useful server for studying evolutionary relationships among whole genomes and exploring information about the OGs used to infer phylogenetic relationships. Although at present it addresses only bacteria, we anticipate that BPhyOG will expand to contain more completely sequenced archaeal and eukaryotic genomes. It may also be useful, though challenging, to allow users to infer variant phylogenies for a set of selected genomes by changing the criteria for homologous comparison.

## Conclusion

BPhyOG is an interactive online server for reconstructing whole genome phylogenies. It allows users to infer phylogenies for any set of genomes of interest to study their evolutionary relationships by visualizing the tree directly on the web, or provides a Newick format treefile for further study. BPhyOG also allows users to retrieve information about OGs, as well as homologous OG pairs, in subtree-specific genomes or the whole set of genomes. The current version (V-1.0) of BPhyOG contains 177 completely sequenced bacterial genomes and 79,855 OG pairs are involved. BPhyOG will be updated once every 1–2 years if the computing power in our laboratory allows.

## Availability and requirements

Project name: BPhyOG

Project home-page: 

Operating system(s): Available as web-based service, accessible via any web-browser

Programming languages: Perl, C, JavaScript and HTML

License: GNU GPL

Any restrictions to use by non-academics: None

## Abbreviations

BPhyOG: Bacterial Phylogenies based on Overlapping Genes

OGs: Overlapping Genes

ORF: Open Reading Frame

NCBI: National Center for Biotechnology Information

BLAST: the Basic Local Alignment Search Tool

MySQL: My Structured Query Language

NJ: Neighbor Joining

UPGMA: Unweighted Pair-Group Method using Arithmetic Averages

PHYLIP: Phylogeny Inference Package

## Authors' contributions

YQL arranged the OG data from NCBI, carried out the phylogeny reconstructions and drafted the manuscript. CF, YQL, DYZ and KL designed the database structure and built the website. KL conceived and coordinated the study, was responsible for the phylogeny-inferring project and helped YQL to draft the manuscript. All authors read and approved the final manuscript.

## Supplementary Material

Additional file 1 The correlation between all ORFs and OG pairs in 177 genomes. As expected, the number of OG pairs in each genome is significantly correlated with its total number of ORFs (Pearson's correlation coefficient is 0.668; P < 0.01). Click here for file

Additional file 2 Two phylogenetic trees for 30 gamma-proteobacteria genomes. (a) The tree is based on 16S rRNA sequences using the NJ method. The 16S rRNA sequences were obtained from the Ribosomal Database Project-II release 9 (RDP) [24]. (b) The tree is based on the number of orthologous OG pairs inferred by the UPGMA method. Click here for file
