# Supplementary figures and images for: BPhyOG: An interactive server for genome-wide inference of bacterial phylogenies based on overlapping genes
Source: BMC Bioinformatics. 2007 Jul 25;8:266. doi: 10.1186/1471-2105-8-266 (PMC1940028; doi:10.1186/1471-2105-8-266)

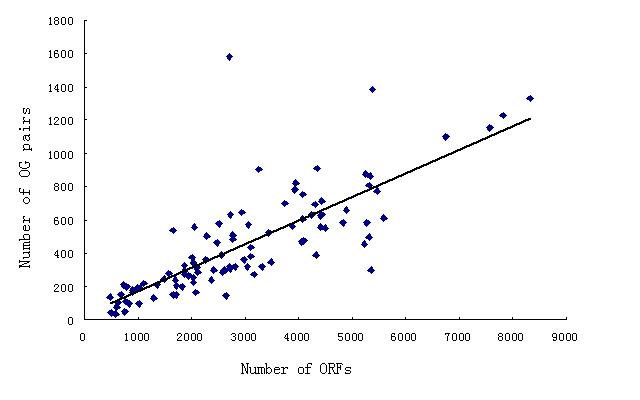

Supplement: Additional file 1 — The correlation between all ORFs and OG pairs in 177 genomes. As expected, the number of OG pairs in each genome is significantly correlated with its total number of ORFs (Pearson's correlation coefficient is 0.668; P < 0.01). [file 1471-2105-8-266-S1.jpeg]

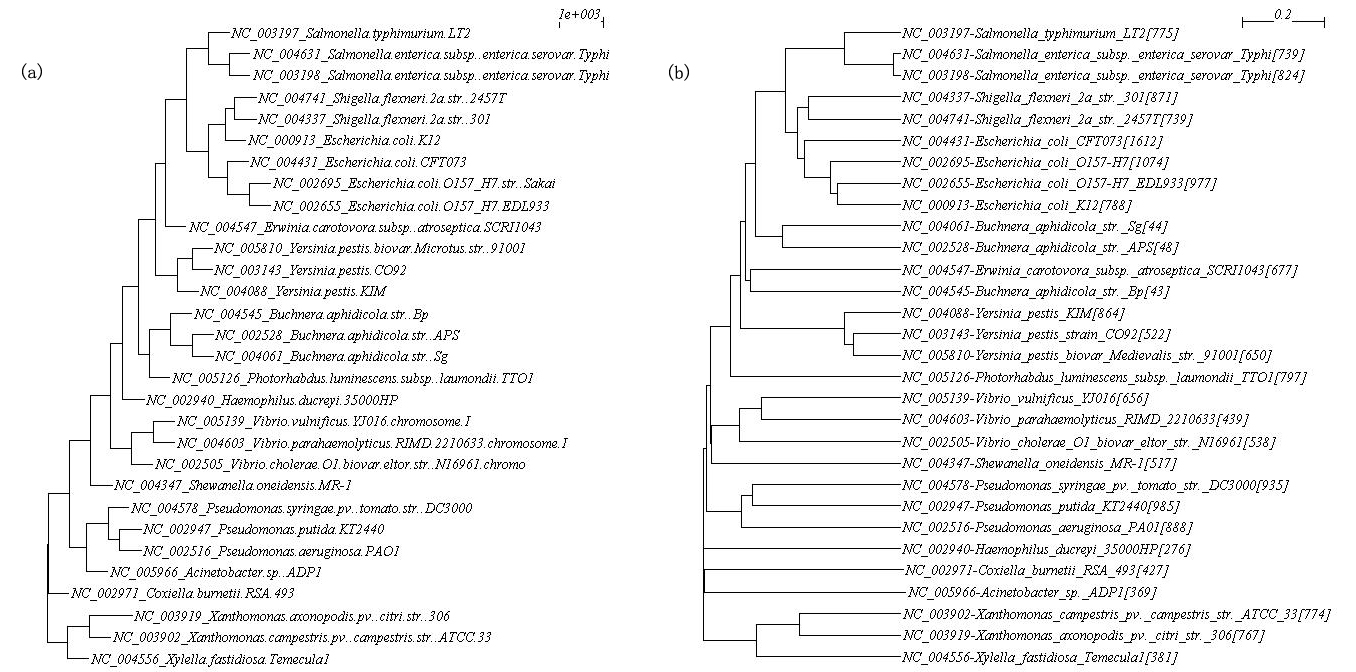

Supplement: Additional file 2 — Two phylogenetic trees for 30 gamma-proteobacteria genomes. (a) The tree is based on 16S rRNA sequences using the NJ method. The 16S rRNA sequences were obtained from the Ribosomal Database Project-II release 9 (RDP) [24]. (b) The tree is based on the number of orthologous OG pairs inferred by the UPGMA method. [file 1471-2105-8-266-S2.jpeg]
